# Gingival Phenotype and Associated Factors in Orthodontically Treated Individuals and Untreated Controls: A Cross-Sectional Comparative Study

**DOI:** 10.3390/jcm15114338

**Published:** 2026-06-03

**Authors:** Resül Çolak, İsmail Gül, Orhan Cicek

**Affiliations:** 1 Department of Periodontics, Faculty of Dentistry, Zonguldak Bülent Ecevit University, Zonguldak 67600, Türkiye; ismail.gul@beun.edu.tr; 2 Department of Orthodontics, Faculty of Dentistry, Zonguldak Bülent Ecevit University, Zonguldak 67600, Türkiye; orhancicek@beun.edu.tr

**Keywords:** gingival phenotype, orthodontic treatment, periodontal health, keratinized gingival width, oral hygiene, periodontal tissues

## Abstract

**Background/Objectives**: Gingival phenotype is considered an important factor influencing periodontal tissue response during orthodontic treatment; however, the association between orthodontic treatment history and gingival phenotype remains unclear. This study aimed to compare gingival phenotype between orthodontically treated individuals and untreated controls and to evaluate the factors associated with gingival phenotype, including periodontal status and oral hygiene habits. **Methods**: This cross-sectional comparative study included 180 individuals aged 18–35 years, who were divided into two groups according to orthodontic treatment history (history of orthodontic treatment, *n* = 90; untreated controls, *n* = 90). Gingival phenotype was assessed using the transparency method at the mid-buccal aspect of the mandibular central incisor. Periodontal parameters, including Plaque Index (PI), Gingival Index (GI), probing depth (PD), and keratinized gingival width (KGW), were recorded. Oral hygiene habits and behavioral factors were also evaluated. Binary logistic regression analysis was performed to identify factors associated with gingival phenotype. Statistical significance was set at *p* < 0.05. **Results**: No significant association was found between orthodontic treatment and gingival phenotype (*p* > 0.05). Periodontal parameters were comparable between groups; however, orthodontically treated individuals demonstrated significantly better oral hygiene habits (*p* < 0.05). Logistic regression analysis revealed that male sex (OR = 6.54, *p* < 0.001) and increased KGW (OR = 1.668, *p* = 0.003) were independently associated with a thick gingival phenotype. **Conclusions**: The findings indicate that gingival phenotype appeared to be more strongly associated with intrinsic anatomical factors, particularly sex and KGW, rather than orthodontic treatment. It was concluded that individualized assessment of gingival phenotype and controlled orthodontic tooth movement may contribute to periodontal preservation.

## 1. Introduction

Oral diseases represent a major public health challenge with significant adverse effects on both individuals and societies [1]. Current epidemiological evidence indicates that dental caries, periodontal diseases, and associated tooth loss affect billions of people worldwide, constituting a substantial global disease burden [2]. These conditions not only result in functional limitations and esthetic concerns at the individual level but also impose considerable economic and organizational pressures on healthcare systems. Therefore, improving our understanding of the etiology of periodontal diseases and developing effective preventive strategies are essential for advancing both clinical practice and public health policies [2,3].

Health and disease are shaped not only by clinical findings but also by individual and behavioral factors; nevertheless, objective clinical parameters remain central to the assessment of periodontal health [4,5,6,7,8]. Periodontal diseases are chronic infectious conditions initiated by pathogenic microorganisms within dental plaque biofilms, while the host response—modulated by genetic, environmental, and local factors—plays a critical role in disease onset and progression [9]. Supragingival plaque accumulation at the gingival margin represents the primary etiological factor for gingivitis; although not all gingivitis progresses to periodontitis, gingival inflammation is a prerequisite for its development [3,10]. Consequently, periodontal diseases are largely preventable and can be effectively controlled through adequate plaque management and patient compliance [9].

The concept of phenotype describes a dynamic condition shaped by both genetic determinants and environmental influences, distinguishing it from the more genetically oriented concept of biotype; accordingly, the 2017 World Workshop on the Classification of Periodontal and Peri-Implant Diseases and Conditions replaced the term “biotype” with “phenotype,” defining it as the three-dimensional morphological characteristics of the periodontal soft and hard tissues [11]. Clinically, a thin phenotype is associated with delicate gingiva, a narrow zone of keratinized tissue, and thin alveolar bone, whereas a thick phenotype presents with denser, more fibrotic gingiva and greater bone thickness [11,12]. This conceptual shift has driven growing interest in gingival phenotype, particularly in relation to its role in periodontal–orthodontic interactions.

Despite its growing clinical relevance, the assessment of the gingival phenotype in routine practice remains challenging due to the lack of a universally accepted and standardized method [13,14]. Several approaches, including transgingival probing, ultrasonic devices, and cone–beam computed tomography (CBCT), have been proposed for this purpose. Among these, the transparency method (TRAN), based on the visibility of a periodontal probe through the gingival margin, is widely used in clinical settings, where probe visibility within the sulcus is indicative of a thin gingival phenotype [15,16,17].

The effects of orthodontic treatment on periodontal health remain a matter of ongoing debate. With the growing number of adult orthodontic patients, maintaining periodontal health has become increasingly important. While orthodontic treatment may improve oral hygiene by correcting dental alignment and reducing plaque-retentive areas, orthodontic appliances can also promote plaque accumulation and hinder effective oral hygiene, potentially leading to gingival inflammation and periodontal tissue alterations. Previous studies have reported increased plaque accumulation and a higher prevalence of gingivitis, particularly in patients treated with fixed appliances [18,19]. In addition, biological and morphological changes in periodontal tissues during orthodontic treatment have been documented, highlighting the need for careful evaluation of tissue responses [20,21]. Consequently, periodontal outcomes during and after orthodontic treatment are influenced by multiple factors, including oral hygiene practices, systemic conditions, host response, and anatomical characteristics of periodontal tissues. In this context, the gingival phenotype has emerged as a key determinant influencing the periodontal tissue response to orthodontic treatment and related biological and mechanical stimuli [22,23].

Although numerous studies have investigated the effects of orthodontic treatment on periodontal tissues [20,21,22,23], gingival phenotype has frequently been evaluated as a secondary variable, while important confounding factors such as oral hygiene habits and periodontal parameters have not always been adequately controlled. Furthermore, evidence examining the relationship between orthodontic treatment history and gingival phenotype together with behavioral factors remains limited [24,25,26]. In addition, a previous study evaluating periodontal changes associated with orthodontic treatment was characterized by methodological limitations, including the absence of an appropriate control group and heterogeneity in study design, which may complicate direct comparisons of the findings [27]. In this context, further well-designed comparative studies are needed to clarify the factors associated with gingival phenotype and their potential clinical implications. A better understanding of these associations may provide clinically relevant insights for both periodontists and orthodontists in the assessment and preservation of periodontal health during orthodontic treatment. To the best of our knowledge, few previous studies have simultaneously evaluated gingival phenotype, periodontal parameters, oral hygiene behaviors, and orthodontic treatment history within a comparative study design, with a specific focus on the mandibular anterior region [24,28,29,30].

Therefore, the present study aimed to compare periodontal status, oral hygiene habits, and gingival phenotype between individuals with and without a history of orthodontic treatment and to evaluate the associations between gingival phenotype and periodontal and behavioral factors. The first hypothesis of the study was that gingival phenotype is associated with orthodontic treatment status, whereas the second hypothesis was that gingival phenotype is associated with periodontal and behavioral factors.

## 2. Materials and Methods

### 2.1. Study Design, Ethics, and Consent

This cross-sectional comparative study was designed to evaluate the association between orthodontic treatment history and gingival phenotype while accounting for potential confounding factors, including oral hygiene status and periodontal inflammation. Ethical approval was obtained from the Non-Interventional Clinical Research Ethics Committee of Zonguldak Bülent Ecevit University (Approval No: 2025/22; Date: 17 December 2025). The study was conducted in accordance with the Declaration of Helsinki, and written informed consent was obtained from all participants.

### 2.2. Sample Size Calculation

Sample size adequacy for the binary logistic regression analysis was evaluated using the events-per-variable (EPV) approach [31,32]. The less frequent outcome category in the present study was the thin gingival phenotype, which included 50 participants. Considering the final multivariable logistic regression model, which retained two independent predictors, the EPV value was 25, exceeding the commonly recommended threshold of EPV ≥ 20 for stable logistic regression estimates [31,32]. Accordingly, the total sample size of 180 participants was considered adequate for the regression analysis performed in this study [31,32].

### 2.3. Eligibility Criteria and Groups

Participants aged 18–35 years were included in this study. All participants were systemically healthy or had controlled systemic conditions not affecting periodontal status and were either periodontally healthy or diagnosed with gingivitis [11]. Participants were allocated into two groups based on orthodontic treatment history.

#### 2.3.1. Orthodontically Treated Group

Completed non-extraction fixed orthodontic treatment at least 12 months prior to periodontal examination;

Treatment limited to cases with mild crowding or diastema (≤3 mm);

Availability of complete orthodontic records and panoramic radiographs [24].

#### 2.3.2. Untreated (Control) Group

No history of orthodontic treatment, confirmed by clinical examination and patient records [24].

#### 2.3.3. Exclusion Criteria (For Both Groups)

Presence of periodontal pockets ≥ 5 mm;

Inadequate restorations or presence of fixed or removable prosthetic appliances;

Crowns or restorations in the mandibular anterior region;

Use of medications known to affect gingival tissues (e.g., calcium channel blockers, immunosuppressants, anticonvulsants);

Presence of parafunctional habits (e.g., bruxism);

Pregnancy or lactation [11].

### 2.4. Clinical and Periodontal Assessments

A comprehensive periodontal examination was performed in all participants to ensure an objective assessment of periodontal status. Gingival phenotype was assessed using the transparency method by inserting a periodontal probe 1 mm into the gingival sulcus and evaluating its visibility through the gingival tissue; visible probe indicated a thin phenotype, whereas non-visibility indicated a thick phenotype. The mid-buccal aspect of the mandibular right central incisor was used as the reference site [28,29,33].

Plaque Index (Silness and Löe) and Gingival Index (Löe and Silness) were recorded at four sites per tooth (mesiobuccal, distobuccal, mid-buccal, and mid-lingual/palatal), and mean values were calculated [34,35]. Probing depth (PD) was measured at four sites per tooth and recorded in millimeters [36], while keratinized gingival width (KGW) was measured at the mid-buccal aspect of each tooth as the distance between the gingival margin and the mucogingival junction [37]. Frenulum tension in the mandibular anterior region was assessed based on the level of frenal attachment and recorded as present or absent [38].

All measurements were performed using a UNC-15 periodontal probe (CPU 15 UNC, Hu-Friedy^®^, Chicago, IL, USA). All periodontal examinations were conducted by a single calibrated periodontist (I.G.) under standardized clinical conditions.

### 2.5. Oral Hygiene and Behavioral Factors

Oral hygiene and behavioral variables, including tooth brushing frequency, dental floss use, smoking status, and frequency of dental visits after orthodontic treatment, were recorded using a standardized data collection form developed in accordance with variables commonly evaluated in previous periodontal and orthodontic clinical studies [19,29]. These variables were assessed through patient interviews and clinical records and were systematically entered into a structured Microsoft Excel database (Microsoft Corp., Redmond, WA, USA) for analysis. The recorded variables were evaluated for their potential association with gingival phenotype and were included as independent variables in the regression analysis.

### 2.6. Orthodontic Treatment Characteristics and Cephalometric and Panoramic Assessment

The orthodontically treated group consisted of individuals who had completed non-extraction fixed orthodontic treatment between 2020 and 2025 at the Department of Orthodontics, Faculty of Dentistry, Zonguldak Bülent Ecevit University. To reduce treatment heterogeneity, only cases with mild crowding or diastema treated with standardized fixed appliance mechanics were included. All orthodontic treatments were performed by a single orthodontist (O.C.) using 0.022 × 0.028-inch MBT^TM^ stainless steel brackets (Mini Master Series, American Orthodontics, Sheboygan, WI, USA) according to the MBT™ prescription. The archwire sequence included heat-activated nickel–titanium round wires during the initial alignment phase, followed by stainless steel round and rectangular archwires, and finalized with 0.019 × 0.025-inch stainless steel rectangular archwires [39]. No maxillary or mandibular expansion protocols were performed in the included cases. Following treatment completion, all individuals received fixed canine-to-canine lingual retainers in both arches using 0.0215-inch six-stranded stainless steel wire. The mean duration of orthodontic treatment was approximately 15.2 ± 2.5 months.

To further characterize mandibular incisor movement patterns in individuals with a history of orthodontic treatment, pretreatment and posttreatment lateral cephalometric radiographs retrospectively retrieved from the departmental archives were evaluated. All lateral cephalometric radiographs had been obtained using the same cephalometric radiographic system (Veraviewepocs 2D, J Morita Mfg. Corp., Kyoto, Japan). Cephalometric analyses were digitally performed by the same orthodontist (O.C.) using the NemoCeph analysis software (NemoStudio v20.10.0; Nemotec S.L., Madrid, Spain). Changes in mandibular incisor inclination and sagittal position were determined by calculating the differences between pre- and posttreatment values of IMPA, L1-NB angle, and L1-NB distance measurements. The L1-NB angle was defined as the angle formed between the long axis of the mandibular central incisor and the NB line, whereas the IMPA represented the angle between the mandibular plane and the long axis of the mandibular incisor. The L1-NB distance was defined as the linear distance between the most labial point of the mandibular incisor crown and the NB line [27].

Panoramic radiographs obtained using the same radiographic system (Veraviewepocs 2D, J Morita Mfg. Corp., Kyoto, Japan) were evaluated during the posttreatment retention period to assess the presence of any tooth loss or alveolar bone loss.

### 2.7. Statistical Analysis

Statistical analyses were performed using SPSS software (IBM SPSS Statistics for Windows, version 27.0; IBM Corp., Armonk, NY, USA). The normality of data distribution was assessed using the Kolmogorov–Smirnov and Shapiro–Wilk tests. Descriptive statistics were expressed as mean ± standard deviation for continuous variables and as frequencies and percentages for categorical variables.

Comparisons between two groups were performed using the Mann–Whitney U test for continuous variables and the chi-square test for categorical variables.

Gingival phenotype was defined as the dependent variable and coded as 0 (thin) and 1 (thick). Binary logistic regression analysis was performed to identify factors associated with gingival phenotype. Variables were selected based on clinical relevance and univariate analysis results, and those with *p* < 0.20 were entered into the multivariable logistic regression model. All clinically relevant periodontal and behavioral variables, including age, oral hygiene behaviors, periodontal parameters, smoking status, dental visits, and orthodontic treatment history, were initially evaluated in the multivariable model. Variables that did not contribute significantly to the model were subsequently excluded to obtain the most parsimonious final model. Multicollinearity among independent variables was assessed using variance inflation factor (VIF) values. The explanatory power of the final model was evaluated using Nagelkerke’s R^2^, and model discrimination was assessed using the area under the curve (AUC). Results were reported as odds ratios with 95% confidence intervals (CIs).

Intra-examiner reliability was assessed by repeating measurements in 25% of randomly selected participants at a two-week interval and evaluated using the intraclass correlation coefficient (ICC), calculated using a two-way random-effects model with absolute agreement. A *p*-value < 0.05 was considered statistically significant.

## 3. Results

Among individuals with a history of orthodontic treatment, the mean change in the L1-NB angle was 2.2 ± 3.4°, with values ranging from −7° to +15°. Similarly, the mean change in the L1-NB distance was 0.7 ± 1.3 mm (range: −2 to +3.8 mm), whereas the mean change in IMPA was 3.4 ± 4.2° (range: −9° to +21°). Overall, the cephalometric findings indicated a mild tendency toward mandibular incisor proclination at the group level; however, as presented in Table 1, both proclination and retrusion movements were observed among individuals with a history of orthodontic treatment.

Intra-examiner reliability was high, with ICC values ranging from 0.707 to 0.969 (*p* < 0.001). The kappa coefficient for gingival phenotype assessment was 0.707, indicating good agreement.

A total of 180 individuals (68 males and 112 females) were included in the study, with 90 participants in each group (treated and untreated). The mean age was 21.07 ± 2.56 years. Gingival phenotype was classified as thin in 50 individuals (27.8%) and thick in 130 individuals (72.2%). The descriptive characteristics of the study population are presented in Table 2.

Comparisons of demographic characteristics, oral hygiene habits, gingival phenotype, and periodontal parameters between individuals with and without orthodontic treatment are presented in Table 3. There was no statistically significant difference between the groups in terms of sex distribution (*p* = 0.124). However, a significant difference was observed for age, with the orthodontically treated group being younger on average (*p* < 0.001).

Regarding oral hygiene habits, individuals with a history of orthodontic treatment had higher frequencies of tooth brushing *(p* < 0.001) and dental floss use (*p* = 0.014). No statistically significant difference was found between the groups in terms of gingival phenotype (*p* = 0.506).

Comparisons of continuous periodontal parameters are presented in Table 4. A significant difference was observed between the groups for age, with the orthodontically treated group being younger (*p* < 0.001). No statistically significant differences were found for probing depth (*p* = 0.109), PI (*p* = 0.084), GI (*p* = 0.235), or KGW (*p* = 0.362).

Factors associated with gingival phenotype are presented in Table 5. In univariate analysis, no statistically significant associations were observed between gingival phenotype and age, frequency of dental visits, tooth brushing frequency, dental floss use, PI, GI, probing depth, or orthodontic treatment status (all *p* > 0.05).

Sex and KGW were significantly associated with gingival phenotype. Males had a significantly higher likelihood of exhibiting a thick phenotype compared to females (OR = 8.46, 95% CI: 3.16–22.68, *p* < 0.001). In addition, each unit increase in KGW was associated with an increased likelihood of a thick phenotype (OR = 1.912, 95% CI: 1.38–2.64, *p* < 0.001).

Frenulum stress was not significantly associated with gingival phenotype (OR = 2.86, 95% CI: 0.94–8.63, *p* = 0.062), although a tendency toward a higher likelihood of a thick phenotype was observed.

In the multivariable binary logistic regression analysis, male sex and greater KGW remained statistically associated with a thick gingival phenotype. Male individuals showed a significantly higher likelihood of a thick phenotype (OR = 6.54, 95% CI: 2.39–17.88, *p* < 0.001), while increasing KGW was also associated with a higher likelihood (OR = 1.668, 95% CI: 1.18–2.34, *p* = 0.003). The final model demonstrated acceptable fit and discrimination (all VIFs < 5, Nagelkerke’s R^2^ = 0.260, AUC = 0.763).

**Figure 1:**
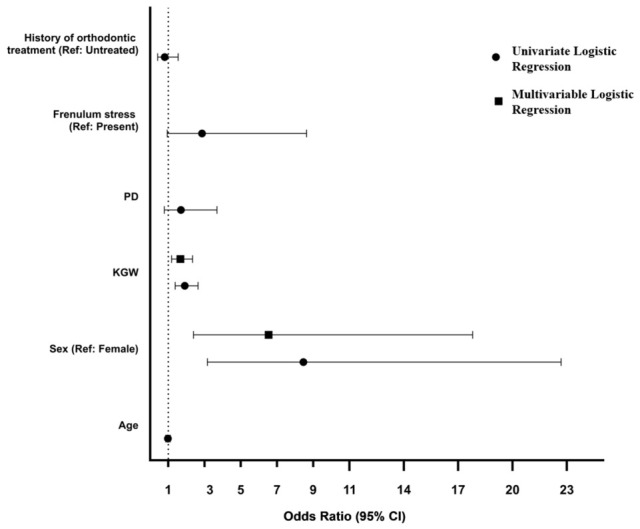
Factors associated with gingival phenotype in univariate and multivariable logistic regression analyses. Odds ratios and 95% confidence intervals (CIs) are shown; the dashed vertical line represents OR = 1; PD: Probing Depth; KGW: Keratinized Gingival Width.

Univariable logistic regression analysis identified several variables potentially associated with gingival phenotype. After adjustment in the multivariable model, male sex and increased KGW remained independently associated with a thick gingival phenotype. The results of the regression analyses are illustrated in Figure 1.

## 4. Discussion

Periodontal tissue changes associated with orthodontic treatment extend beyond esthetic and functional outcomes and involve complex biological adaptation processes. Orthodontic tooth movement induces morphological changes in the alveolar bone, periodontal ligament, and surrounding soft tissues, and these responses are influenced not only by the magnitude and direction of orthodontic forces but also by individual tissue characteristics, including the gingival phenotype [40,41,42]. Despite increasing recognition of its clinical relevance, the role of gingival phenotype in modulating soft tissue responses to orthodontic forces remains incompletely understood, with limited available evidence [11,12,43]. Most previous studies have evaluated gingival phenotype as a secondary or associated factor, rather than as a primary variable of interest. Furthermore, data focusing specifically on the mandibular anterior region are scarce, with the majority of studies primarily addressing gingival recession [24,28,30]. In addition, comparative studies evaluating individuals with and without a history of orthodontic treatment within the same population, while simultaneously considering oral hygiene behaviors, remain limited [30,44,45].

The findings of the present study suggest that gingival phenotype is predominantly associated with intrinsic anatomical and periodontal characteristics rather than orthodontic treatment history alone. While the first hypothesis proposing an association between gingival phenotype and orthodontic treatment status was not supported, the second hypothesis was partially confirmed, as gingival phenotype demonstrated associations with specific periodontal and behavioral determinants. In particular, KGW and sex-related differences appeared to play a more substantial role in phenotype variation than a previous history of orthodontic treatment. These observations underscore the multifactorial nature of gingival phenotype and highlight the importance of simultaneously considering anatomical, periodontal, and behavioral variables when evaluating periodontal risk in orthodontic patients.

Gingival phenotype is influenced by a complex interplay of genetic, anatomical, and environmental factors, including alveolar bone thickness, KGW, tooth morphology and position, as well as age and sex [11,14,46,47]. Previous studies have reported that orthodontic tooth movements performed within biological limits do not significantly affect gingival phenotype or lead to gingival recession [48,49]. In contrast, movements exceeding alveolar bone boundaries, particularly in the labial direction, have been associated with an increased risk of gingival thinning and gingival recession [50,51].

Consistent with these findings, the present study did not demonstrate a significant association between orthodontic treatment history and gingival phenotype. This observation is in agreement with the findings of Dridi et al., [52] who also reported no relationship between orthodontic treatment status and gingival phenotype, identifying root morphology as the primary influencing factor. However, other studies have emphasized the importance of gingival phenotype as a risk indicator in orthodontic treatment planning [26,53,54]. For instance, Musilli et al. [53] highlighted, through the Orthodontic–Periodontal Risk Assessment (OPRA) model, that gingival phenotype plays a critical role in treatment-related risk, particularly when tooth movement exceeds alveolar bone limits [53]. The discrepancies observed among studies may be attributed to differences in orthodontic mechanics, including the direction and magnitude of tooth movement, as well as their relationship with alveolar bone boundaries. In this context, performing tooth movements within alveolar limits may partially explain the absence of a significant association between orthodontic treatment and gingival phenotype observed in the present study.

Previous studies have shown that individuals who have undergone orthodontic treatment tend to exhibit better oral hygiene compared to those without such treatment. In particular, Demirovic et al. [55] reported improved oral hygiene levels among orthodontically treated individuals, a finding that has been consistently supported by subsequent studies [56,57,58]. In line with these observations, the present study also demonstrated that individuals with a history of orthodontic treatment exhibited more favorable oral hygiene habits. This observation may reflect the influence of regular follow-up visits and repeated oral hygiene instructions commonly provided during orthodontic treatment. In addition, individuals seeking orthodontic care may demonstrate greater awareness of oral health and more favorable oral hygiene behaviors [59]. Furthermore, untreated malocclusions may complicate plaque control, while less frequent dental visits may contribute to poorer oral hygiene practices in individuals without orthodontic treatment history. Taken together, these findings suggest a possible association between orthodontic treatment history and improved oral hygiene behaviors.

The relationship between sex and gingival phenotype remains inconsistent in the literature. While some studies have reported no significant differences between males and females [60], others have demonstrated a higher prevalence of a thick gingival phenotype in males [33,61]. In the present study, males were also more likely to exhibit a thick gingival phenotype, supporting the latter findings. This finding suggests that sex may represent an important factor associated with gingival phenotype, potentially reflecting underlying differences in tissue characteristics between males and females. These differences may be attributed to variations in factors such as ethnicity, age distribution, measurement techniques, and periodontal status across study populations.

In the present study, increased KGW was significantly associated with a thick gingival phenotype, suggesting that KGW may represent an important morphological component of the phenotype. This finding is supported by several studies reporting that a narrow band of keratinized tissue is commonly associated with a thin phenotype [60,62,63,64]. However, because KGW is closely related to the clinical characterization of gingival phenotype itself, the observed association should not be interpreted as a completely independent causal relationship. Rather, KGW may represent an anatomically interconnected component of gingival phenotype expression. Nevertheless, as emphasized in the consensus report by Jepsen et al. [11], keratinized gingiva should be considered an integral component in the comprehensive assessment of gingival phenotype.

The present findings should be interpreted in light of several methodological considerations inherent to the study design. The inclusion of only young adult participants may limit the generalizability of the results to adolescents, older adults, and other age groups. In addition, due to the cross-sectional nature of the study, causal relationships between orthodontic treatment history and gingival phenotype cannot be established. Since baseline periodontal and morphological characteristics prior to orthodontic treatment were unavailable, it cannot be determined whether orthodontic treatment altered gingival phenotype over time. Therefore, the findings should be interpreted as associations related to orthodontic treatment history rather than direct treatment effects. Future studies including broader age ranges, large sample sizes, and additional parameters such as alveolar bone thickness are needed to provide a more comprehensive understanding of the relationship between orthodontic treatment and periodontal outcomes. Furthermore, the gingival phenotype in the mandibular anterior region has been reported to be thinner than in other regions and therefore more susceptible to mucogingival problems [16,65]. Although this region is considered more prone to early periodontal alterations, its systematic evaluation as a reference site remains limited.

In the present study, the mandibular central incisor was selected as the reference tooth because of its susceptibility to early periodontal changes and its potential to provide a sensitive clinical assessment site. This methodological approach highlights the potential value of mandibular central incisors as a reference area in periodontal and orthodontic evaluation, while not diminishing the importance of studies focusing on other dental regions, particularly esthetic areas. Nevertheless, the present findings should be interpreted as site-specific observations derived from a single mandibular incisor region and may not fully represent the gingival phenotype characteristics of the entire dentition. In addition, the method used to assess gingival phenotype is consistent with current literature and represents a non-invasive and widely accepted clinical approach [28,29,33]. The use of a calibrated examiner and the high intra-examiner agreement further support the reliability of the measurements.

Since buccal alveolar bone thickness and tooth movement relative to alveolar housing were not directly assessed in the present study, conclusions regarding alveolar boundary-related periodontal risk should be interpreted cautiously. In addition, CBCT imaging was not available; therefore, detailed evaluation of buccal alveolar bone morphology and potential labial bone dehiscence was not possible. From a clinical perspective, the present findings emphasize the importance of individualized periodontal evaluation during orthodontic treatment planning. Although no significant association between orthodontic treatment history and gingival phenotype was observed, carefully controlled orthodontic tooth movement and consideration of periodontal characteristics remain important for periodontal preservation. Given the limitations associated with routine radiographic assessment of alveolar bone thickness, including radiation exposure concerns and current imaging guidelines [66,67], interdisciplinary collaboration between orthodontists and periodontists may contribute to more comprehensive risk assessment and more predictable periodontal outcomes.

## 5. Conclusions

Based on the findings of the present study, the gingival phenotype assessed at the mandibular central incisor region appeared to be more strongly associated with intrinsic anatomical and periodontal characteristics than with orthodontic treatment history alone. Although orthodontic treatment status was not associated with gingival phenotype, individuals with previous orthodontic treatment demonstrated more favorable oral hygiene habits; however, this observation may also reflect differences in health awareness, behavioral factors, or follow-up patterns between groups.

In addition, KGW and biologically based sex-related differences were identified as important factors associated with gingival phenotype. These findings emphasize the importance of individualized periodontal assessment during orthodontic treatment planning. In addition, carefully controlled orthodontic tooth movement may contribute to periodontal preservation and minimization of treatment-related risks. Furthermore, interdisciplinary collaboration between orthodontists and periodontists may contribute to more predictable periodontal outcomes during orthodontic treatment.

## Figures and Tables

**Table 1:** Cephalometric changes in the group with a history of orthodontic treatment.

Variable	Mean ± SD	Median	Min–Max
ΔL1-NB angle (°)	2.2 ± 3.4	2.0	−7 to +15
ΔL1-NB distance (mm)	0.7 ± 1.3	0.6	−2 to +3.8
ΔIMPA (°)	3.4 ± 4.2	4.0	−9 to +21

Positive values indicate proclination/protrusion, whereas negative values indicate retroclination/retrusion. SD, standard deviation; Min–Max, minimum–maximum values; Δ, change between pre- and post-treatment measurements; mm, millimeter; °, degree.

**Table 2:** Descriptive characteristics of the study population.

Variable	Sample (*n*:180)	Percentage (%)
Sex		
Female	112	62.2
Male	68	37.8
Frequency of tooth brushing		
Regular	126	70.0
Irregular	54	30.0
Dental floss use		
Not using	157	87.2
Using	23	12.8
Dentist visit		
Irregular	154	85.5
Regular	26	14.5
Gingival phenotype		
Thick	130	72.2
Thin	50	27.8
Frenulum stress		
Absent	166	92.2
Present	14	7.8
History of orthodontic treatment		
Treated	90	50.0
Untreated	90	50.0

*n*: sample; %: percentage.

**Table 3:** Comparison of demographic, behavioral, and periodontal characteristics between untreated and orthodontically treated individuals.

Variable	Untreated (*n* = 90), *n* (%)	Treated (*n* = 90), *n* (%)	*p*
Sex			
Male	39 (43.3)	29 (32.2)	0.124
Female	51 (56.7)	61 (67.8)	
Frequency of tooth brushing			
Irregular	39 (43.3)	15 (16.7)	<0.001 *
Regular	51 (56.7)	75 (83.3)	
Dental floss use			
Not using	84 (93.3)	73 (81.1)	0.014 *
Using	6 (6.7)	17 (18.9)	
Dentist visit			
Irregular	81 (90.0)	73 (81.1)	0.090
Regular	9 (10.0)	17 (18.9)	
Gingival phenotype			
Thin	23 (25.6)	27 (30.0)	0.506
Thick	67 (74.4)	63 (70.0)	
Frenulum stress			
Absent	83 (92.2)	83 (92.2)	1.000
Present	7 (7.8)	7 (7.8)	

Chi-square test; *p* < 0.05, *: *p* < 0.05, *n*: sample, %: percentage.

**Table 4:** Comparison of continuous periodontal parameters between individuals with and without a history of orthodontic treatment.

Variable	Untreated (*n* = 90)	Treated (*n* = 90)	*p*
	Mean ± SD (Median)	Mean ± SD (Median)	
Age	21.76 ± 2.68 (21)	20.39 ± 2.25 (20)	<0.001 *
PD	1.69 ± 0.53 (1.5)	1.79 ± 0.30 (1.8)	0.109
PI	1.84 ± 0.79 (2)	2.07 ± 0.95 (2)	0.084
GI	1.17 ± 0.84 (1)	1.31 ± 0.83 (1)	0.235
KGW	3.29 ± 0.88 (3)	3.17 ± 1.45 (3)	0.362

Mann–Whitney U test; PD: Probing Depth; PI: Plaque Index; GI: Gingival Index; KGW: Keratinized Gingival Width; SD: Standard deviation; *n*: sample; *p*: significance level, *: *p* < 0.05.

**Table 5:** Factors associated with gingival phenotype (binary logistic regression analysis).

Variable	Univariate Logistic Regression			Multivariable Logistic Regression		
	OR	95% CI	*p*	OR	95% CI	*p*
Age	0.978	0.86–1.11	0.726			
Sex (Ref: Female)	8.46	3.16–22.68	<0.001 *	6.54	2.39–17.88	<0.001 *
KGW	1.912	1.38–2.64	<0.001 *	1.668	1.18–2.34	0.003 *
PD	1.70	0.78–3.69	0.183			
Frenulum stress (Ref: Present)	2.86	0.94–8.63	0.062			
History of orthodontic treatment (Ref: Untreated)	0.80	0.41–1.54	0.506			

OR: Odds Ratio; 95% CI: 95% Confidence Interval; PD: Probing Depth; KGW: Keratinized Gingival Width; *p*: significance level, *: *p* < 0.05.

## Data Availability

The data that support the findings of this study are available from the corresponding author upon reasonable request.

## Abbreviations

The following abbreviations are used in this manuscript:

PI	Plaque Index
GI	Gingival Index
PD	Probing Depth
KGW	Keratinized Gingival Width
OR	Odds Ratio
95% CI	95% Confidence Interval
CBCT	Cone–beam Computed Tomography
TRAN	Transparency Method
VIF	Variance Inflation Factor
AUC	Area Under the Curve
ICC	Intraclass Correlation Coefficient
